# Cutaneous symptoms such as acneform eruption and pigmentation are closely associated with blood levels of 2,3,4,7,8-penta-chlorodibenzofurans in Yusho patients, using data mining analysis

**DOI:** 10.1186/1756-0500-2-27

**Published:** 2009-02-25

**Authors:** Tomoaki Imamura, Shinya Matsumoto, Manabu Akahane, Yoshiyuki Kanagawa, Soichi Koike, Bunichi Tajima, Shiro Matsuya, Hiroshi Uchi, Satoko Shibata, Masutaka Furue

**Affiliations:** 1 Nara Medical University, School of Medicine. Department of Public Health, Health Management and Policy, Kashihara, Japan 840 Shijo-cho, Kashihara, Nara 634-8521, Japan; 2 Department of Planning Information and Management, The University of Tokyo Hospital, Tokyo, Japan; 3 Nara Medical University, School of Medicine. Department of Public Health, Health Management and Policy, Kashihara, Japan; 4 Teradata Japan, Ltd., Tokyo, Japan; 5 Medical Ontology Research Unit, Department of Planning Information and Management, The University of Tokyo Hospital, Tokyo Japan; 6 Department of Dermatology, Graduate School of Medical Sciences, Kyushu University, Fukuoka, Japan

## Abstract

**Background:**

Yusho an intoxication caused by oral dioxins and polychlorinated biphenyls occurred in 1968. Patients suffered from various systemic symptoms, including general fatigue, nausea, muscular and articular pain, acneform eruptions, black comedones, cutaneous and oral pigmentation, and increased eye discharge. The major causative factor was the contamination of rice oil with 2,3,4,7,8-penta-chlorodibenzofuran (PeCDF). Recent technical advances have allowed us to measure blood levels of PeCDF. However, there is little information on which symptoms and laboratory data are directly associated with PeCDF levels.

**Methods:**

Yusho patients underwent annual medical check-ups from 2001 to 2003. Blood PeCDF levels were correlated with the presence or absence of symptoms in medical, hematological, dermatological, dental and ophthalmological examinations. This study analyzed all combinations by using the association analysis. This is the most suitable method to evaluate all combinations of the data comprehensively. This method was used to determine the rate of patients with high PeCDF level in the population with each symptom, and to extract combinations of three symptoms which were strongly associated with high PeCDF level.

**Results and Conclusion:**

The rate of the patients with high PeCDF level was high in populations with high uric acid, black comedones (face), second highest quartile of age, or high urea nitrogen. The combination of three symptoms associated with the highest rate of patients with high PeCDF level was "high uric acid, female sexuality, and history of acneform eruptions", followed by "history of Yusho in and after 1968, high cholesterol level, and subjective symptoms." This analysis newly suggested that PeCDF concentration may be associated with history of dermatological symptoms, high uric acid, and elevated erythrocyte sedimentation rate.

## Background

A mass food poisoning involving at least 1900 individuals occurred in northern Kyushu of Japan in 1968. The poisoning was called Yusho (oil disease) because it was caused by ingestion of rice bran oil which was contaminated with Kanechlor-400, a commercial, Japanese brand of polychlorinated biphenyls (PCBs). It was later found that the rice bran oil had been contaminated not only with PCBs, but also with various dioxins. Among these PCB-related compounds, 2,3,4,7,8-penta-chlorodibenzofuran (PeCDF), a highly toxic dioxin, was considered to be the major causative factor [1-5]. The World Health Organization re-evaluated the toxic equivalency factors (TEFs) for seven polycholorinated dibenzo-p-dioxins, 10 polychlorinated dibenzofurans and 12 coplanar PCBs. TEFs for 2,3,4,7,8-PeCDF and 2,3,3',4,4',5-hexachlorobiphenyl (PCB 156) are 0.3 and 0.00003, respectively, compared to 1.0 for the most toxic dioxin, 2,3,7,8-tetracholorodibenzo-p-dioxin [6].

Non-specific subjective symptoms such as general fatigue, weight loss and anorexia were observed in most patients [7]. In addition to these general symptoms, various characteristic objective symptoms gradually appeared in patients, including dermatological symptoms (comedones, acneform eruptions, black spots in hair pores, and dark-brown pigmentation of skin and nails), ophthalmological symptoms (increased cheesy secretions from meibomian glands, conjunctival pigmentation, cysts of meibomian glands and edema of the eyelids), and oral symptoms (gingival pigmentation). A considerable number of patients also suffered from headaches, paresthesia of the extremities, abdominal pain, cough and sputum, altered menstruation, and small-for-date babies. Jaundice and palpable spleen were not observed [1,8-10]. At the time of the outbreak, blood levels of PeCDF were estimated to be as high as > 60,000 pg/g lipids [11]. However, due to technical limitations, blood levels of PeCDF have not been routinely measured until recently. It was started to examine the blood levels of PeCDF in 2001 and found that PeCDF levels were still significantly high in Yusho patients compared with normal controls. Accordingly, we amended the diagnostic criteria for Yusho in 2004 by adding an item of "abnormal blood level of PeCDF" (Table 1). A PeCDF blood level of ≥50 pg/g lipids was considered abnormally high compared to that in normal controls. More than 35 years have elapsed since the outbreak of Yusho and the specific symptoms in Yusho patients have gradually disappeared. However, no studies have addressed the direct relationship/association between PeCDF blood levels and clinical/laboratory symptoms.

**Table 1:** Diagnostic criteria for Yusho (as presently supplemented)

The diagnostic criteria for Yusho were revised on October 26, 1972; supplemented on June 14, 1976; and an item related to blood polychlorinated quarterphenyl (PCQ) level was added on June 16, 1981. The study group of Yusho started to measure blood levels of dioxins in annual medical check-ups from 2001. It was considered appropriate to add an item corresponding to the blood 2,3,4,7,8-penta-chlorodibenzofurans (PeCDF) level; therefore the criteria were supplemented and further revised on September 29, 2004.

Conditions of onset
1) Ingestion of Kanemi rice bran oil contaminated with PCBs,
2) Vertical PCB transmission from mothers with Yusho to infants in some cases,
3) Familial occurrence seen in many cases

Important manifestations
1. Acneform eruptions
Black comedones seen on the face, buttocks and other intertriginous sites; comedones with inflammatory manifestations; and subcutaneous cysts with atheroma-like contents that tended to suppurate.;
2. Pigmentation
Pigmentation of the face, palpebral conjunctivae, gingivae, and nails etc. (including so-called 'black babies');
3. Hypersecretion of the meibomian glands;
4. Unusual composition and concentration of PCBs in the blood;
5. Abnormal level of blood PCQ
1) ≥ 0.1 ppb: an abnormally high concentration,
2) 0.03 to 0.09 ppb: the boundary between high and normal concentrations,
3) ≤ 0.02 ppb (detection limit): normal concentration;
6. Abnormal level of blood PeCDF
1) ≥ 50 pg/g lipids: an abnormally high concentration,
2) 30 to 50 pg/g lipids: a relatively high concentration,
3) < 30 pg/g lipids: normal concentration]

Standard symptoms and findings
1. Subjective symptoms
1) General fatigue,
2) Headaches, dull headaches,
3) Paresthesia of the extremities (abnormal sensation),
4) Increased eye discharge,
5) Cough and sputum,
6) Constant abdominal pain,
7) Altered menstruation
2. Objective findings
1) Manifestation of bronchitis,
2) Deformation of nails,
3) Bursitis,
4) Increased neutral fat in the serum,
5) Increased serum γ-glutamyl transpeptidase (γ-GTP),
6) Decrease in serum bilirubin,
7) Small-for-date baby,
8) Growth retardation and dental abnormality (retarded eruption of permanent teeth)

With recent technical advances in the measurement of dioxins such as PeCDF, it has become possible to measure blood PeCDF levels during routine annual medical check-ups in Yusho patients. Since 2001, measurement of blood PeCDF level has been carried out not only in Yusho patients (determined patients), but also in persons who had not yet been determined as having Yusho (undetermined cases) [12,13]. Therefore, it is now possible to determine which symptoms and laboratory abnormalities are actually related to PeCDF blood levels. Although routine logistic regression analyses and analyses of variance have been conducted many times, the results demonstrated that the associations between PeCDF and clinical symptoms did not completely correspond with the impressions of medical practitioners who were actually engaged in the diagnosis. When combinations of symptoms characteristic for PeCDF were extracted as a trial, it was pointed out that combinations corresponding with the impressions of medical practitioners became available. The procedure that allowed the most efficient extraction of combinations of characteristic symptoms was selected to conduct more detailed analyses. For this high-capacity data analysis, we took advantage of recent data mining techniques.

## Methods

### Symptoms and laboratory examinations performed in Yusho patients during annual medical check-ups

Following the outbreak of Yusho, medical check-ups for more than 1900 Yusho-determined patients were conducted at follow-up in several prefectures for > 30 years. Yusho medical check-ups consisted of a total of 241 items, including 55 interview and physical items, 21 dermatological, five ophthalmological, 108 dental, and 52 laboratory examinations [1,13,14]. Medical data from 477 patients who underwent Yusho medical check-ups from 2001 to 2003 were selected for this study. Subjective symptoms were general fatigue, headache, cough, sputum, abdominal pain, diarrhea, constipation, numbness, arthralgia, and altered menstruation. Physical examinations included body height, body weight, pulse rate, blood pressure, nutrition, heart sounds, respiratory sounds, hepatomegaly, splenomegaly, edema, lymphadenopathy, as well as tendon reflexes, sensory examination, chest radiography, electrocardiography, and abdominal ultrasonography. Dermatological symptoms evaluated included black comedones, acneform eruptions, scar formation, pigmentation of skin and nails, and nail deformity. Ophthalmological symptoms evaluated included abnormal eye discharge, edema of the eyelid, conjunctival pigmentation, meibomian gland cysts, and cheesy secretions from meibomian glands. Dental and oral manifestations evaluated included gingivitis, marginal periodontitis, retarded eruption of permanent teeth, tooth pigmentation, odontogenesis imperfecta, abnormal occlusion, and pigmentation of gingival mucosa, buccal mucosa, palate and lips. Laboratory examinations included blood levels of PeCDFs, complete blood cell counts, erythrocyte sedimentation rate, blood chemistry, blood hepatitis B antigen, and alfa-fetoprotein. Sex, quartile age, and information on determined/undetermined status were also used in the analyses.

### Methods of data analysis

This study analyzed all combinations by using association analysis [15]. This is the most suitable method to evaluate all combinations of the data comprehensively. This method was used to determine the proportion of high level PeCDF in the population with each symptom, and to extract combinations of three symptoms which were strongly associated with high PeCDF level. Logistic-regression analysis can be used to evaluate the links between factors and a disease. However, this is a method to evaluate the linkage with only one such factor. Therefore, this is not suitable, for example, to find three primary symptoms. In contrast, association analysis is the most suitable method to evaluate combinations of the data comprehensively. Association analysis is also known as the term "market basket analysis," which analyzes a tendency that some items are bought with others.

We used the association analysis as a method to find out the three symptoms [16]. This study determined the proportion of the patients with high PeCDF level in the population with each symptom, and extracted combinations of three symptoms which were strongly associated with high PeCDF level.

## Results

### Rate of the patients with high PeCDF level in the population with each symptom

Table 2 shows rate of the patients with high PeCDF level in the population with each symptom. The rate was highest in the population with black comedones (trunk), followed by the population with high uric acid. However, the black comedone (trunk) was not statistically significant, because the rate of patients with the symptom was too low. In contrast, high uric acid was statistically significant, because the rate of patients with the symptom was adequate. High uric acid was followed by black comedones (face), third quartile of age, and high urea nitrogen.

**Table 2:** Rate of patients with high PeCDF level in population with each symptom

**Rank**	**Symptoms**	**Rate of patients with the symptom**	**Rate of patients with high PeCDF (> 50 pg/g lipids)**
**1**	Black comedones (trunk) <+ or severer>	0.0671	0.6563
**2**	uric acid (high)	0.1761	0.6310*
**3**	Black comedones (face) <+ or severer>	0.0860	0.6098
**4**	Third quartile age	0.2348	0.6071*
**5**	urea nitrogen (high)	0.1111	0.6038
**6**	red blood cell count (low)	0.1342	0.5938
**7**	History of pigmentation <present>	0.3669	0.5886*
**8**	Classification of testee <qualified>	0.7526	0.5794*
**9**	Subjective symptoms, etc. <present>	0.1488	0.5775
**10**	Second quartile age	0.2788	0.5714

* Statistically significant symptoms

### Rate of patients with high PeCDF level in the population with each three-symptom combination

Table 3 shows 10 populations with three-symptom combinations having the highest rate of patients with high PeCDF level. In the population with high uric acid, female sexuality, and history of acneform eruptions, 88.89% of the patients had high PeCDF level. This is the highest rate among all combinations of three symptoms. The second highest rate was seen in the combination of history of Yusho in and after 1968, high cholesterol level, and presence of subjective symptoms. The 10 combinations listed in the table 3 were all statistically significant.

**Table 3:** Rate of patients with high PeCDF level in each population with three symptoms

	**Symptom 1**	**Symptom 2**	**Symptom 3**	**Proportion of patients with the symptoms**	**Proportion of patients with high PeCDF (> 50 pg/g lipids)**
**1**	uric acid (high)	Sex <female>	History of acneform eruptions <present>	0.0755	0.8889
**2**	History of Yusho in and after 1968 <present>	cholesterol (high)	Subjective symptoms, etc. <present>	0.0692	0.8485
**3**	uric acid (high)	1-hour erythrocyte sedimentation rate (elevated)	History of acneform eruptions <present>	0.0545	0.8462
**4**	uric acid (high)	1-hour erythrocyte sedimentation rate (elevated)	Sex <female>	0.0650	0.8387
**5**	Subjective symptoms, etc. <present>	Sex <female>	History of pigmentation <present>	0.0755	0.8333
**6**	uric acid (high)	Sex <female>	History of pigmentation <present>	0.0629	0.8333
**7**	cholesterol (high)	Heaviness of head/headache <present>	Subjective symptoms, etc. <present>	0.0503	0.8333
**8**	History of Yusho in and after 1968 <present>	Subjective symptoms, etc. <present>	Second quartile age	0.0503	0.8333
**9**	History of Yusho in and after 1968 <present>	uric acid (high)	1-hour erythrocyte sedimentation rate (elevated)	0.0713	0.8235
**10**	1-hour erythrocyte sedimentation rate (elevated)	Subjective symptoms, etc. <present>	Sex <female>	0.0713	0.8235

## Discussion

The mean blood level of PeCDF in patients with Yusho was 11.6–16.8 times higher than the mean levels in normal controls. It is known that dioxins and PCBs remain in tissues for a long time. It is of note that even 40 years after the outbreak, high concentrations of dioxins remained in the blood of patients with Yusho.

Many studies have been conducted on Yusho, however, few have reported analysis on symptoms and PeCDF levels. There are two main reasons for this: (i) PeCDF measurements include large errors; and (ii) no distinction can be made between specific symptoms and changes due to aging, since more than 40 years have passed since the occurrence. Therefore, routine logistic regression analysis cannot clarify the relationship between the symptoms and PeCDF levels.

The level of PeCDF was high in patients with black comedones (trunk). However, since there were few patients with the symptom, the association was not judged as statistically significant. The symptom judged as statistically significant was high uric acid. Although diagnostic criteria for Yusho do not include the uric acid, the uric acid level was high in elderly Yusho patients. It might be a symptom that has been manifested recently.

A population with three-symptom combination showing high rate of patients with high PeCDF level tends to have (1) female sexuality, (2) dermatological symptoms incorporated in the diagnostic criteria such as histories of "acneform eruptions" and "pigmentation," and (3) symptoms such as "high uric acid" and "elevated erythrocyte sedimentation rate." Although "high uric acid" is a symptom strongly associated with males, it was seen linked with females. The symptom may be enhanced due to the aging of the patients.

Principal component analyses have revealed that PeCDF level is strongly associated with PCB and polychlorinated quarterphenyl (PCQ) levels, and also associated with blood glucose, arthralgia, total cholesterol, urine sugar, 2-hour erythrocyte sedimentation rate, thymol, and sodium as well as conventionally known dermatological symptoms such as acneform eruptions and black comedones, and total bilirubin.

Nearly 40 years have elapsed since the outbreak of Yusho, and the patients have become old. Differentiating Yusho symptoms from alterations due to aging is difficult, but the analysis suggested that PeCDF level may be associated with the history of dermatological symptoms, high uric acid, and elevated erythrocyte sedimentation rate.

## Competing interests

The authors declare that they have no competing interests.

## Authors' contributions

TI designed the study and drafted the manuscript. SM designed the data analysis, analyzed data, and assisted manuscript drafting. MA, YK and SK assisted manuscript drafting. BT analyzed data. SM assisted designing of data analysis. TU and SS collected data. MF designed the whole study and assisted manuscript drafting. All the authors, except MA and TK, reviewed the final manuscript and gave approval.
